# Deer Hunting Season and Firearm Violence in US Rural Counties

**DOI:** 10.1001/jamanetworkopen.2024.27683

**Published:** 2024-08-14

**Authors:** Patrick Sharkey, Juan Camilo Cristancho, Daniel Semenza

**Affiliations:** 1 Princeton University School of Public and International Affairs, Princeton, New Jersey; 2 School of Education, University of California, Irvine; 3 Department of Sociology, Anthropology, and Criminal Justice, Rutgers University, Camden, New Jersey; 4 Department of Urban-Global Public Health, Rutgers University, Camden, New Jersey; 5 New Jersey Gun Violence Research Center, Rutgers University, Camden

## Abstract

**Importance:**

Firearm violence is a major public health problem in the US. However, relatively little research has focused particular attention on firearm violence in rural areas, and few studies have used research designs that draw on exogenous variation in the prevalence of firearms to estimate the association between firearm presence and shootings.

**Objective:**

To investigate the association between the start of deer hunting season and shootings in rural counties in the US.

**Design, Setting, and Participants:**

In this cohort study, data from all rural US counties in states with available data on the timing of deer hunting season were matched with data on shootings from the Gun Violence Archive from January 1, 2014, to December 31, 2021.

**Exposure:**

Shootings in the first 3 weeks of deer hunting season were compared with the week prior to the start of deer hunting season.

**Main Outcomes and Measures:**

The main outcome was daily total shootings. The association between the start of deer hunting season and shootings was estimated using Poisson regression models to analyze change within counties while controlling for relevant calendar year, month of year, and seasonal effects.

**Results:**

The sample included 854 rural counties with a mean (SD) population of 16 416 (18 329) per county and 5.4 (13.3) annual shootings per 100 000 people. The county fixed-effects specification analyzing the association between deer hunting season and shootings showed that relative to the week prior to deer hunting season, the incidence rate ratio for total shootings was 1.49 (95% CI, 1.13-1.95) for the first week of deer hunting season and 1.41 (95% CI, 1.02-1.94) for the second week of deer hunting season. Estimates remained consistent when excluding hunting accidents and were most pronounced in states with more hunting licenses per capita.

**Conclusions and Relevance:**

In this cohort study of the association between the start of deer hunting season and firearm violence, results showed that the start of deer hunting season was associated with a substantial increase in shootings. The findings highlight the role of firearm prevalence in gun violence and suggest the need for focused policies designed to reduce firearm violence in areas with substantial hunting activity during the first weeks of deer hunting season.

## Key Points

**Question:**

What is the association between the start of deer hunting season and shootings in rural US counties?

**Findings:**

This cohort study examined variation within 854 rural US counties in the timing of the start of deer hunting season, in which modern firearms are used. The first week of deer hunting season was associated with a significant increase in shootings relative to the week prior.

**Meaning:**

This study suggests that the start of deer hunting season is associated with a substantial increase in shootings, highlighting the role of firearm prevalence in gun violence.

## Teaser

This cohort study investigates the association between the start of deer hunting season and the number of shootings in rural counties in the US.

## Introduction

Firearm injuries and deaths are a significant public health problem in the US.^1,2^ A large body of research suggests that greater availability of firearms is associated with increased risk for firearm injury, accidental shootings, suicide, and homicide in the home, across US states, and among high-income nations throughout the world.^3,4,5,6^ This research has been predicated on the premise that the prevalence of guns in a given home, community, or nation is likely to make violent incidents more injurious and more deadly.^7,8,9,10,11,12^ However, to our knowledge, few studies have been designed to exploit plausibly exogenous variation in the prevalence of firearms in public and private settings to generate evidence on the association between firearm prevalence and shootings.

This study adds new evidence to the literature on firearm presence and gun violence by using variation in the timing of deer hunting season across US counties. Throughout much of the rural US, the start of deer hunting season is a major annual event that leads to an abrupt increase in the number of people with firearms in public and private spaces.^13,14,15,16,17,18,19,20^ The timing of deer hunting season, or the date when residents can begin hunting deer with modern firearms, varies across states and, in some cases, across counties within states. This variation provides a natural experiment for assessing whether the opening of deer hunting season is associated with the number of shootings in rural US counties.

## Methods

This study integrates several data sources spanning from January 1, 2014, to December 31, 2021, a period for which we collected data on both shootings and deer hunting season start dates. First, we created a county-level dataset using the date that deer hunting season starts and its duration. States typically have distinct seasons for different types of hunting. We focused on the earliest date that hunters can begin using modern firearms to hunt deer, which generally matches with the period with the largest deer harvest. The vast majority of hunters hunt big game, and deer hunting is by far the most common form of big game hunting.^14,17^ For example, between one-fifth and one-third of the total annual deer harvested in Wisconsin are killed in the opening weekend of deer hunting season, and 70% of annual deer harvested are killed in the first 9 days of the season.^14^ One study conducted in rural counties found that the percentage of male arrestees armed with a long gun increased 300% at the start of deer hunting season, providing evidence that the deer hunting season is associated with a sharp increase in the presence of firearms in public and private spaces.^14^ The study was determined to be exempt from review by Princeton University’s institutional review board because it is not human participants research. The study adheres to the Strengthening the Reporting of Observational Studies in Epidemiology (STROBE) reporting guideline for cohort studies.

Due to the absence of a centralized source for information on deer hunting season and variation within states, we coded the start of deer hunting season using diverse references, including state departments of fish and wildlife reports, hunting magazines, and newspaper notices. Dates were reported at the state level or by state-defined hunting zones, which often align with counties. We excluded all counties in Arizona, Iowa, Louisiana, Nevada, New Mexico, and Wyoming because it was not possible to match counties to specific deer hunting seasons due to within-state variation in deer hunting season start dates and misalignment between counties’ borders and hunting zones. The group of excluded states includes some with relatively high (Iowa and Wyoming), medium (Louisiana, New Mexico, and Utah), and low (Arizona) rates of hunting licenses per capita; thus, there does not seem to be a clear link between the prevalence of hunting in a state and the complexity of the timing and geographic variation in hunting seasons. Nonetheless, it is unlikely that the methods exploiting variation in the timing and location of deer hunting season can be used in these states, and our results pertain only to rural counties outside of these 6 states.

The second source of data is the Gun Violence Archive (GVA), which documents shootings across the US, including information about the incident, location, date, type of gun, and number of victims.^21^ Gun Violence Archive data are based on various published sources, including media and government reports and local police department reports. Each recorded shooting is geostamped and includes the date of the incident, allowing us to merge each incident with our county dataset by location and timing. The GVA includes a field indicating if the incident was a hunting accident, based on media or police reports. A growing number of studies draw on data from the GVA because it is the only source that tracks all shootings across the entire country.^22^ Research validating data from the GVA has found that total counts of homicides correspond closely with data collected and published by the Centers for Disease Control and Prevention.^22^ However, recent work has shown that the GVA may undercount certain types of incidents, with a bias toward shootings with multiple victims and those involving women and children.^23,24^

**Figure 1.**
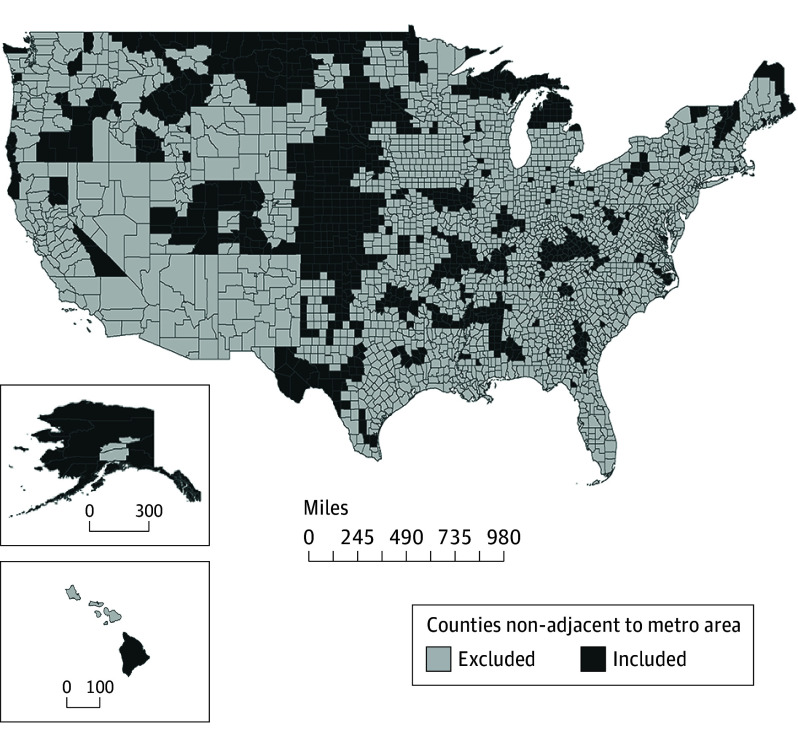
Distribution of Counties Included in the Analytical Sample There were 854 counties included in the analytical sample. Eligibility criteria included states with complete data on deer hunting season start dates and counties not in or adjacent to a metropolitan area. To convert miles to kilometers, multiply by 1.6.

To create our sample, we classified all counties according to the National Center for Health Statistics urban-rural classification.^25^ Research on the prevalence of hunting with firearms shows that participation is extremely low (and often not legal) in counties that are part of or near metropolitan areas and increases substantially in sparsely populated areas outside of central cities and suburbs.^14^ For this reason, we reduced the final sample to include 854 counties not located in metropolitan areas and not adjacent to a metropolitan area. Figure 1 shows the location of counties included in the analysis. In the last step, we merged each state with the number of hunting licenses per capita, collected from the US Fish and Wildlife Service between 1980 and 2016.^10^

### Statistical Analysis

The statistical analysis exploits variation in the precise timing of the start of deer hunting season across and within US counties to identify the association between deer hunting season and gun violence. Although in some states deer hunting season lasts longer than 3 weeks, data on deer harvests suggest that the number of deer killed typically increases sharply in the first week of the season, subsides by week 2, and decreases quickly in the weeks afterward. All models use a Poisson regression in which the outcome is the count of the number of total shootings on a specific day in the specified county.^26^ We ran the same set of models using negative binomial regressions, and the results were extremely similar. All SEs adjust for heteroskedasticity and clustering at the county level. We report 95% CIs for all point estimates. We use 2-sided *z* score tests to determine statistical significance. Although we report 95% CIs for all estimates, we refer to results as statistically significant if *P* < .05. Statistical analysis was performed with Stata/SE, version 2018.0 (StataCorp LLC).

The sequence of models begins with a specification that draws on variation across counties (equation 1 in eAppendix 1 in Supplement 1). The model includes indicators for each 7-day period before and after the start of deer hunting season, ranging from 3 weeks prior to the start of the season to the third week of the season. We included fixed effects for the calendar year, the day of the week (dummy indicators for Sunday through Saturday, with Monday as the reference), and 2 indicators for holidays—one for the period including the Wednesday to Sunday before and after Thanksgiving, and another for the period from December 23 through January 2.

We then estimated a second model including county fixed effects (equation 2 in eAppendix 1 in Supplement 1). In this second model, we compared the weeks after the start of deer hunting season with the weeks prior to deer hunting season within the same counties.^27,28^ We used the same controls as in model 1. In a third model, we altered the outcome to exclude shootings labeled in the GVA as hunting accidents. This model allowed us to assess whether the association between deer hunting season and shootings was due simply to accidents occurring while hunting, or whether any association was instead due to shootings other than hunting accidents.

Last, we examined how results vary by the prevalence of hunting across states. Using a measure of the number of hunting licenses per capita, we grouped states into tertiles representing states with relatively low, middle, or high prevalences of hunting. We conducted the main analyses separately for each category of states to assess whether results were stronger in states with more hunting licenses per capita.

## Results

**Figure 2.**
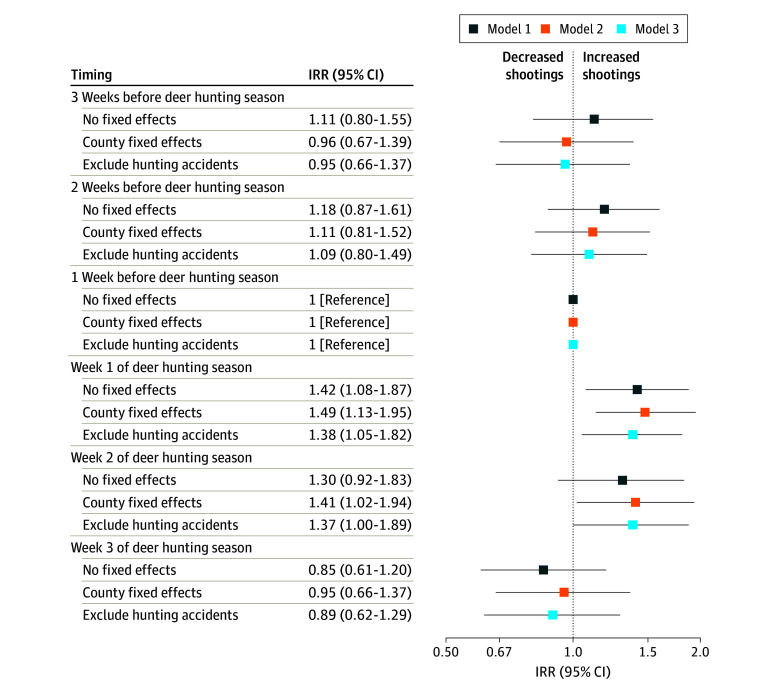
Association Between Deer Hunting Season and Shootings Incidence rate ratios (IRRs) from Poisson regression. Model 1 is a Poisson regression with year, month of year, and day of week fixed effects, plus indicators for the Wednesday to Sunday before and after Thanksgiving and the period from December 24 to January 2. Models 2 and 3 include county fixed effects. The outcome for models 1 and 2 include all shootings. The outcome for model 3 excludes all hunting accidents. Standard errors are adjusted for heteroskedasticity and clustering at the county level. Error bars indicate 95% CIs.

Figure 2 displays results from models focusing on total shootings for the 854 rural counties (mean [SD] population, 16 416 [18 329] per county; 5.4 [13.3] annual shootings per 100 000 people) in our analytical sample. Of the 854 counties in the sample, 305 had at least 1 shooting during the study period. The graphs show results from 3 models: the first comparing weeks before and after deer hunting season when pooling all counties together, the second making the same comparisons within counties (county fixed effects), and the third showing the county fixed-effects results after excluding hunting accidents. The similarity of results between the first 2 models suggests that findings are robust to the decision to make comparisons across vs within counties. Focusing on the left side of the graph, incidence rate ratios for weeks prior to the start of deer hunting season were close to zero and were not statistically significant, indicating no clear trend in shootings before the start of deer hunting season. This finding provides evidence that the timing of deer hunting season can be considered exogenous.

The point estimates shown in “Week 1 of deer hunting season” capture the association between the first week of deer hunting season and total shootings, relative to the week before deer hunting season (Figure 2). The county fixed-effects specification shows that the incidence rate ratio was 1.49 (95% CI, 1.13-1.95) for the first week of deer hunting season and 1.41 for the second week of deer hunting season (95% CI, 1.02-1.94). There was no statistically significant association in the third week of deer hunting season.

The third set of models in Figure 2 excludes all shootings labeled as hunting accidents from the outcome measure. Results from this model were close to identical to the results including all shootings, with incidence rate ratios slightly smaller in magnitude. In this model, the incidence rate ratio was 1.38 (95% CI, 1.05-1.82) for the first week of deer hunting season and 1.37 (95% CI, 1.00-1.89) for the second week of deer hunting season.

**Figure 3.**
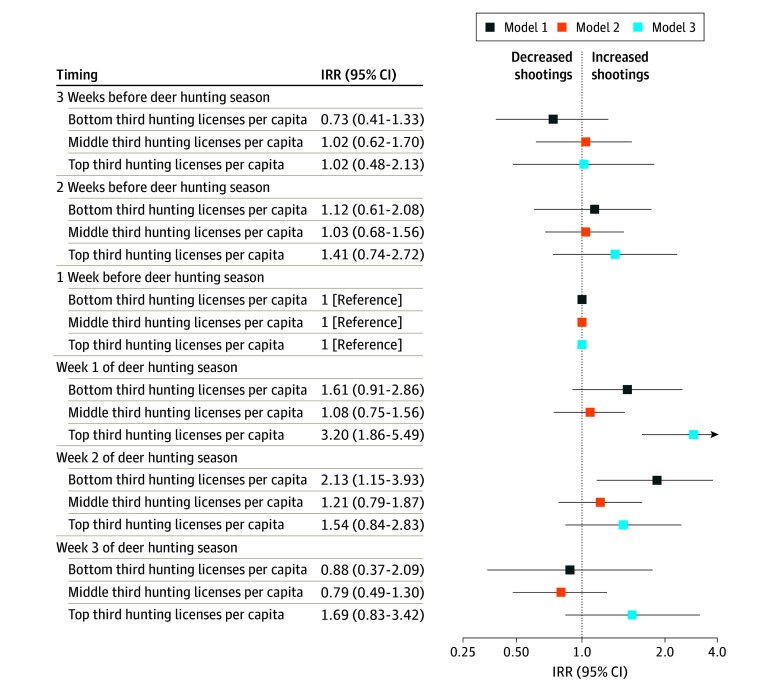
Association Between Deer Hunting Season and Shootings, by State Prevalence of Hunting Licenses per Capita Incidence rate ratios (IRRs) from Poisson regression. All models are county fixed-effects Poisson regressions with year, month of year, and day of week fixed effects, plus indicators for the Wednesday to Sunday before and after Thanksgiving and the period from December 24 to January 2. The outcome for all models includes all shootings. Standard errors are adjusted for heteroskedasticity and clustering at the county level. Error bars indicate 95% CIs.

Figure 3 shows results from county fixed-effects specifications estimated among states in the lowest third, middle third, and highest third of hunting licenses per capita. Reducing the sample of states led to less precise estimates, and we did not find a linear association between hunting licenses per capita and shootings. We did find, however, that the strongest association between deer hunting season and total shootings was in states with the highest number of hunters relative to the population. Among states in the top third of licenses per capita, the incident rate ratio for shootings in the first week of deer hunting season was 3.19 (95% CI, 1.86-5.49).

We conducted a set of additional tests of robustness, results of which are available in eAppendix 2 and the eFigure in Supplement 1. First, we conducted an analysis that included information on the type of gun used in each shooting incident. We found that the increase in shootings after the start of deer hunting season was more pronounced for shootings with a handgun rather than a long gun. However, this finding should be interpreted with caution, as data on the type of gun used are incomplete for a large percentage of incidents in the GVA data. Second, we conducted a supplemental analysis mirroring the main results but focusing only on fatal shootings, rather than total shootings. Full model results are shown in eAppendix 2 and the eFigure in Supplement 1. Results from our preferred county fixed-effects specification were extremely similar to models of all shootings; the incidence rate ratio for fatal shootings was 1.57 (95% CI, 1.11-2.21) for the first week of deer hunting season and 1.44 (95% CI, 0.98-2.10) for the second week of deer hunting season.

## Discussion

We analyzed the association between the start of deer hunting season and shootings in 44 states between 2014 and 2021. Our analysis produced 3 key findings. First, in our preferred models examining variation within counties, we found significantly higher rates of shootings in the first week after the start of deer hunting season relative to the week prior to deer hunting season. The magnitude of the association was smaller in the second week of deer hunting season and was close to zero by the third week. Second, the results were largely replicated when hunting accidents were removed from the analysis. Third, the association of deer hunting season with shootings was most pronounced in states with the greatest number of hunting licenses per capita. Taken together, these findings offer evidence that the start of deer hunting season has a strong association with gun violence. In the sample of rural US counties, more people were killed by gunfire in the first week of deer hunting season than in any other week of the calendar year.

Although the core finding from the analysis is clear, there are multiple ways to interpret this finding. One plausible interpretation is that the beginning of deer hunting season leads to more people walking through the woods with guns, which is associated with an increase in accidental shootings relative to the weeks before deer hunting season. Although it is true that the start of deer hunting season brings about an increase in hunting-related accidental shootings, these shootings are rare, as most hunting accidents do not involve firearms.^16,17^ Models excluding hunting accidents produced results that were nearly identical to the main results, suggesting that our estimates were not due to accidental shootings.

A second plausible interpretation is that the association between deer hunting season and gun violence is due to an unrelated change that occurs at the same time as the beginning of deer hunting season. The main analysis shows clearly that the exact timing of deer hunting season, which varies from state to state and in some cases from county to county, is associated with a sharp change in shootings, providing evidence that it is associated with deer hunting season instead of some other change occurring around the same time. To bolster this evidence, we stratified states by the number of hunting licenses per capita and demonstrated that the findings are much stronger in states with a large hunting population.

A third plausible interpretation is that the findings are associated with the start of deer hunting season but due to behavioral shifts unrelated to firearms, such as people being out in public more often, sleeping less, taking time off from work to hunt, or consuming more alcohol. These behavioral changes might increase opportunities for shootings to take place and create conditions for interpersonal conflicts to turn violent. We are not able to fully rule out these mechanisms with our data; however, 1 prior study showed that the start of deer hunting season was associated with null effects on overall crime, as measured with data reported by police departments, as well as a reduction in alcohol-related arrests, suggesting that the start of deer hunting season is not causally related to broader patterns of alcohol-related behavior or more generalized criminal activity.^14^ At the same time, the same study found that the number of male arrestees armed with a long gun in rural jurisdictions increased 300% with the opening of deer hunting season, suggesting that the prevalence of firearms around hunting season increases dramatically.^14^

This evidence leads us to conclude that the most plausible explanation for the increase in shootings the week after the start of deer hunting season is the heightened presence of firearms in public and private spaces. This conclusion is supported by the supplemental analysis showing that the increase in shootings after the start of deer hunting season was more pronounced for shootings with a handgun rather than a long gun. Although the data on type of gun are incomplete for a large percentage of incidents, this additional evidence suggests the main results may be associated with a broader influx of guns into public and private spaces, rather than simply an increase in long guns used for hunting.^29,30,31^ It will be helpful for future research to incorporate different methods and sources of data, including qualitative data on gun prevalence during hunting season and in other parts of the year, to disentangle these explanations.

### Limitations

There are limitations to the present study that provide opportunities for future research. First, we excluded 6 states from our analysis due to incomplete data about hunting season start dates across the years of the analysis; any conclusions should be drawn only about the states in our sample. Second, our study relies on shooting data from a single source, the GVA. Data from GVA have been shown to have a bias toward incidents that receive more media attention and do not include comprehensive counts of firearm suicides.^23,24^ Considering that much of the literature on the prevalence of guns has focused on suicide, we consider this a natural extension of our research and encourage similar analyses with alternative data sources that provide details on the location and timing of suicides. Third, we analyzed a relatively small analytic sample given our focus on counties not in or adjacent to metropolitan areas. Although this decision is appropriate for studying the association between hunting season and gun violence, future research should seek similar approaches to studying shocks that lead to more guns in public and private spaces in more densely populated areas where most shootings take place.

## Conclusions

Our findings align with a body of research showing that firearm prevalence is associated with an increase in the risk of firearm violence. Although our findings do not address state or federal policy, research showing that state-level firearm regulations reduce shootings is relevant to discussions of how states might respond.^7^ States with more firearm regulations, especially policies such as waiting periods and background checks, have lower overall firearm death rates, including both homicide and suicide.^7,32^ Enhanced firearm regulations that govern firearm storage, carrying, and purchasing, particularly in states where deer hunting is popular, may serve to reduce the number of shootings that occur at the onset of the hunting season.^4,7,12,32^

## Notes

Supplement 1. **eAppendix 1.** Analysis **eAppendix 2.** Results From Models of Fatal Shootings **eFigure.** Association Between Deer Season and Fatal Shootings

Supplement 2.
Data Sharing Statement
