# Supplementary material for: Deer Hunting Season and Firearm Violence in US Rural Counties
Source: JAMA Netw Open. 2024 Aug 14;7(8):e2427683. doi: 10.1001/jamanetworkopen.2024.27683 (PMC11325211; doi:10.1001/jamanetworkopen.2024.27683)
Supplement: Supplement 1. — eAppendix 1. Analysis eAppendix 2. Results From Models of Fatal Shootings eFigure. Association Between Deer Season and Fatal Shootings [file jamanetwopen-e2427683-s001.pdf]

Supplementary Online Content

Sharkey P, Cristancho JC, Semenza D. Deer hunting season and firearm violence in US rural counties. *JAMA Netw Open.* 2024;7(8):e2427683. doi:10.1001/jamanetworkopen.2024.27683

eAppendix 1. Analysis
eAppendix 2. Results From Models of Fatal Shootings
eFigure. Association Between Deer Season and Fatal Shootings

This supplementary material has been provided by the authors to give readers additional information about their work.

© 2024 Sharkey P et al. *JAMA Network Open.*

eAppendix 1. Analysis

The analysis in Figure 2 of the main text begins with the Poisson regression model shown in Equation 1. The outcome λ_{ic} is the count of the number of total shootings occurring on day *i* in county *c*; $D_{ic}$is a set of indicators for each seven day period before and after the start of deer season, ranging from three weeks prior to the start of deer season (*t*=1) to the third week of deer season (*t*=6):

$$\ln(\lambda_{ic}) = \beta_0 + \sum_{t=1}^{T} \beta_{ic} D_{ic} + Day_{ic} + Year_{ic} + Holiday_{ic} + e_{ic} \qquad (1)$$

The week prior to the start of deer season serves as the reference period and the coefficients for the indicators for each week of deer season represent the association between deer season and total shootings. We included fixed effects for the calendar year, the day of the week, and two indicators for holidays—one for the period including the Wednesday-Sunday before and after Thanksgiving, and another for the period running from December 23rd through January 2nd. All standard errors adjust for heteroskedasticity and clustering at the county level.

Results from the first model make comparisons among all counties pooled together. For a more precise comparison, we estimated a second model including county fixed effects, represented by ζ_t in Equation 2:

$$\ln(\lambda_{ic}) = \beta_0 + \sum_{t=1}^{T} \beta_{ic} D_{ic} + Day_{ic} + Year_{ic} + Holiday_{ic} + \zeta_c + e_{ic} \qquad (2)$$

In this model we compared the weeks after the start of deer season to the weeks prior to deer season *within the same counties.* We used the same controls and adjustments for clustering at the county level. We estimated additional analyses that included county by year fixed effects, in

which comparisons were made within the same counties in the same years. Results were nearly identical to the county fixed effects specification shown in Equation 2, and are available upon request. The third model reported in Figure 2 of the main text used an alternative outcome excluding shootings labeled as hunting accidents. This model allowed us to assess whether the identified relationship between deer season and shootings was driven simply by accidents occurring while hunting, or whether the relationship was instead driven by shootings other than hunting accidents.

© 2024 Sharkey P et al. *JAMA Network Open.*

eAppendix 2. Results From Models of Fatal Shootings

In addition to our main model exploring the total number of shootings as the outcome, we present supplemental results for fatal shootings. Results are displayed in eFigure. Because there are many fewer rural counties that have fatal shootings in the brief window before and after the start of deer season, this analysis draws on variation from a much smaller group of counties; for this reason we prefer the analysis of all shootings for our main analysis. However, we include these supplemental results because fatal shootings is an outcome of particular interest to many readers.

We found that results were extremely similar to models of all shootings presented in Figure 2. Focusing on results from the county fixed effects specifications (the middle columns in eFigure), results show that the incidence rate ratio for fatal shootings was 1.57 (CI = 1.11 to 2.21) for the first week of deer season, and 1.44 (CI = .98 to 2.10) for the second week of deer season. There was no statistically significant association in the third week of deer season. In the third column of results, we again found that excluding hunting accidents from the outcome measure did not change results in a meaningful way. In this model, the incidence rate ratio for fatal shootings was 1.49 (CI = 1.05 to 2.11) for the first week of deer season and 1.42 (CI = .98 to 2.07) for the second week of deer season.

© 2024 Sharkey P et al. *JAMA Network Open.*

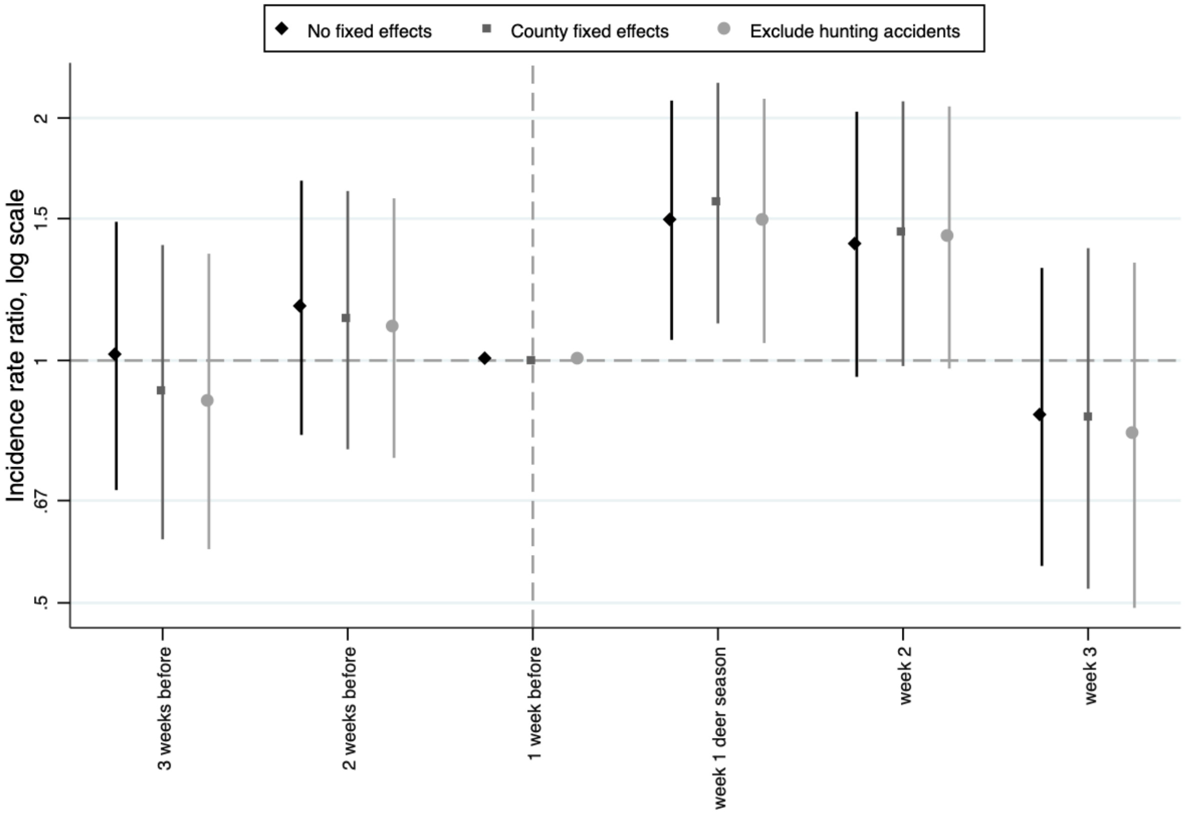

eFigure. Association Between Deer Season and Fatal Shootings.

Notes: Y axis = incidence rate ratio from poisson regression, with the week before the start of deer season as the reference. Model 1 is a poisson regression with year, month of year, and day of week fixed effects, plus indicators for the Wednesday-Sunday before and after Thanksgiving and the period from December 24-January 2. Models 2 and 3 include county fixed effects. The outcome for Models 1 and 2 includes all fatal shootings. The outcome for Model 3 excludes all hunting accidents. Standard errors are adjusted for heteroskedasticity and clustering at the county level. Error bars represent 95% confidence intervals.

© 2024 Sharkey P et al. *JAMA Network Open.*
