# Supplementary material for: Deer Hunting Season and Firearm Violence in US Rural Counties
Source: JAMA Netw Open. 2024 Aug 14;7(8):e2427683. doi: 10.1001/jamanetworkopen.2024.27683 (PMC11325211; doi:10.1001/jamanetworkopen.2024.27683)
Supplement: Supplement 2. — Data Sharing Statement [file jamanetwopen-e2427683-s002.pdf]

Data Sharing Statement

Sharkey. Deer Hunting Season and Firearm Violence in US Rural Counties. *JAMA Netw Open*. Published August 14, 2024. doi:10.1001/jamanetworkopen.2024.27683

Data
Data available: Yes
Data types: Data (not involving human participants)
How to access data: https://dataverse.harvard.edu/dataverse/sharkey
When available: With publication

Supporting Documents
Document types: Statistical/analytic code
How to access documents: https://dataverse.harvard.edu/dataverse/sharkey
When available: With publication

Additional Information
Who can access the data: Anyone requesting the data
Types of analyses: any purpose
Mechanisms of data availability: without investigator support
